# Regional Projections of Extreme Apparent Temperature Days in Africa and the Related Potential Risk to Human Health

**DOI:** 10.3390/ijerph121012577

**Published:** 2015-10-12

**Authors:** Rebecca M. Garland, Mamopeli Matooane, Francois A. Engelbrecht, Mary-Jane M. Bopape, Willem A. Landman, Mogesh Naidoo, Jacobus van der Merwe, Caradee Y. Wright

**Affiliations:** 1 Natural Resources and the Environment Unit, Council for Scientific and Industrial Research, Pretoria 0001, South Africa; E-Mails: matooanemr@yahoo.com (M.M.); FEngelbrecht@csir.co.za (F.A.E.); m.m.bopape@reading.ac.uk (M.-J.M.B.); WALandman@csir.co.za (W.A.L.); MNaidoo4@csir.co.za (M.N.); JHvdMerwe@csir.co.za (J.M.); cwright@mrc.ac.za (C.Y.W.); 2 Unit for Environmental Sciences and Management, North West University, Potchefstroom 2520, South Africa; 3 School of Geography, Archaeology and Environmental Studies, University of the Witwatersrand, Johannesburg 2000, South Africa; 4 Meteorology Department, University of Reading, Reading RG6 6BB, UK; 5 Department of Geography, Geoinformatics and Meteorology, University of Pretoria, Pretoria 0028, South Africa

**Keywords:** climate change, human health, Africa, regional climate modelling, climate services

## Abstract

Regional climate modelling was used to produce high resolution climate projections for Africa, under a “business as usual scenario”, that were translated into potential health impacts utilizing a heat index that relates apparent temperature to health impacts. The continent is projected to see increases in the number of days when health may be adversely affected by increasing maximum apparent temperatures (AT) due to climate change. Additionally, climate projections indicate that the increases in AT results in a moving of days from the less severe to the more severe Symptom Bands. The analysis of the rate of increasing temperatures assisted in identifying areas, such as the East African highlands, where health may be at increasing risk due to both large increases in the absolute number of hot days, and due to the high rate of increase. The projections described here can be used by health stakeholders in Africa to assist in the development of appropriate public health interventions to mitigate the potential health impacts from climate change.

## 1. Introduction

The world’s climate is rapidly changing, largely as a result of human activities that have released large amounts of greenhouse gases (GHG) into the atmosphere. These increased emissions of GHGs have led to changes in the Earth’s climate, including warming at the Earth’s surface. Africa has already experienced increasing warming trends (*i.e.*, increasing maximum and minimum temperatures), and it is likely that surface temperatures across Africa will increase at a rate faster than the projected global average increases in surface temperatures [1,2]. These current and projected increases in temperature have the potential to directly impact human health across the continent.

High ambient temperatures affect the body’s thermoregulatory system, leading to the body’s inability to maintain thermal balance [3]. Exposure to high temperatures can lead to physical disorders including discomfort, fatigue, and heat stroke, and may also lead to death [4]. Many epidemiological studies have demonstrated an association between excess high temperature and increased mortality from cardiovascular, cerebrovascular and respiratory diseases, as well as increases in all-cause mortality [3,5,6,7,8,9,10,11,12,13,14,15,16,17]. The elderly, young children, and people with compromised health status and/or pre-existing diseases are especially vulnerable to negative health impacts from exposure to high temperatures [3]. Additionally, negative health impacts have been shown in some studies to be heightened in urban areas [3]. Urban areas may be more vulnerable for numerous reasons, including the urban island effect, which would intensify local temperatures, as well as the presence of high levels of air pollution, which can have many of the same negative health effects as those from exposure to high temperatures [3].

Physiologically, when the temperature of the human body rises above 38 °C, heat exhaustion can occur, and heat stroke is possible above 40.6 °C [18]; however, exposure to much lower ambient temperatures than these body temperatures may also have large health impacts, ranging from respiratory and cardiovascular effects, to death. The temperature–mortality relationship follows a non-linear, U, J or a V shape, with mortality excesses at low temperatures and high temperatures, and a minimum in mortality at an “optimal” temperature [19,20,21]. The heat threshold can be considered the temperature at which the negative effects of heat on health begin [21,22]. In general, mortality from high temperatures increases rapidly and non-linearly at temperatures above those associated with minimal mortality [19,20,21]. The majority of research on quantifying the relationship between temperature and mortality has occurred in industrialized nations and in temperate climates; there are very few studies in developing countries in general, and Africa specifically [13,18]. Previous studies in northern Ghana, Nairobi, Kenya, and northwest Burkina Faso have found linkages between increased ambient temperatures and mortality, though the associations vary by age, gender and, in some cases, the cause of death [15,16,17,23]. For example, in two informal settlements in Nairobi, increases in mean temperature above the 75th percentile mean temperature led to a statistically significant (at the 95% confidence interval) increase in mortality for children 4 years old and under, as well as an increase in mortality associated with non-communicable diseases (classified in this study as cancers, diabetes, hypertension and other) [16]. In the Kasena-Nankana Districts in northern Ghana, a statistically significant association between all-cause mortality and mean daily temperatures above the 75th percentile (used as the threshold temperature) were observed [17]. At lag days 0–1, a 1.14% increase (95% confidence interval: 0.12%, 1.54%) in all-cause mortality was observed with a 1 °C increase in mean temperatures above the 75th percentile mean daily temperature [17]. Thus, despite the warmer climate, the health of populations in Africa has been found to be negatively impacted by high temperatures.

As a result of climate change, heat-related health impacts may worsen in the future due to projected temperature increases [18]. The magnitude of the future temperature-related health impacts may, however, be offset by acclimatization [22]. Adaptation measures, including behavioral change and implementation of effective heat response plans and interventions, may assist populations to acclimatize to living with high temperatures [24]. The degree of acclimatization through these measures may however be limited, as physiologically there is a limit to the temperatures that humans can survive, and technological interventions such as air conditioning would need large amounts of power, and would be inaccessible or unaffordable to many populations [25]. In fact, very little is known on the rate at which populations can acclimatize to changing temperatures [11,26]. There is some evidence that a heat wave which occurs earlier in the season results in higher mortality than one that occurs later in the season; this might suggest that populations can acclimatize to higher temperatures over a season [6,8,27,28]. However, these seasonal variations do not represent acclimatization to new temperature regimes [26]. Vigotti *et al.* [29] suggest that acclimatization to a new climate regime can may not occur in a lifetime, as they found that an adult’s heat tolerance is influenced by the climate of their birthplace. Previous studies in industrialized countries have found evidence of decreasing heat-related mortality rates in the past century [30,31,32,33,34,35]. There could be multiple reasons for these decreases, including increased adaptation due to factors such as technological improvements, building and urban design improvements, improvements in the health sector, behavioral or physiological adaptation, and increased awareness of the risks to health from high temperatures, as well as factors unrelated to adaptation (e.g., changes in causes of death, demographic factors) [30,31,32,33,34,35].

Little is known on the potential impacts of high temperatures on human health across Africa. Since there is the potential for relatively large increases in temperatures in Africa due to climate change, and there are a high number of vulnerable populations in the region, it is imperative to understand the potential temperature-related health impacts in the region. Mapping of the potential risk of health to increasing temperatures will assist in identifying the geographical areas that may be “hot spots” for negative impacts to human health from projected increases in temperature. This information could then aid in the development of appropriate preventative measures. Regional climate modelling can provide high resolution climate projections for areas, and thus begin to spatially resolve the variability in risk across the continent. Regional modelling tailored for specific areas is particularly important given the many and varied factors that may modify how temperatures affect people’s health in the region including high levels of poverty, high disease burden, and high vulnerability to climate extremes, as well as the spatial variability of these vulnerabilities and the spatial variability of the projected changes in climate. This study aimed to (1) describe the potential risks to human health from high temperatures as a result of climate change across Africa; and (2) highlight to health stakeholders the types of climate services that could be used to assist in the development of appropriate mitigation and preventative plans and measures.

## 2. Materials and Methods

Six high-resolution regional climate model simulations over Africa were obtained using the Conformal Cubic Atmospheric Model (CCAM), for the period 1961–2100 under the A2 (low mitigation, “business as usual”) emission scenario of the Special Report on Emission Scenarios (SRES) used in the Fourth Assessment Report (AR4) of the Intergovernmental Panel on Climate Change (IPCC) [36,37,38,39]. Emission scenarios are used in climate change modelling to develop projections of one potential climate future. Emission scenarios prescribe a GHG emissions pathway for the future, and then climate models use this emissions pathway to project the future climate.

CCAM is a variable resolution global atmospheric model that was developed by the Commonwealth Scientific and Industrial Research Organisation (CSIRO) [36,37]. As a first step, the CCAM was run with a quasi-uniform resolution of about 200 km over the whole globe using sea-surface forcing from the following coupled global climate models: CSIRO, GFDL20 (Geophysical Fluid Dynamic Laboratory), GFDL21 (Geophysical Fluid Dynamic Laboratory), MIROC (Model for Interdisciplinary Research on Climate), MPI (Max Planck Institute for Meteorology) and UKMO (United Kingdom Met-Office). The CCAM used each of the six models’ sea surface forcings separately in order to develop a set of six projections of future climate; this is referred to as the six ensemble members. In order to downscale these global projections to regional projections over Africa (*i.e.*, produce projections over a smaller spatial domain at a higher spatial resolution), the CCAM was then applied in stretched-grid mode, nudged within the 200 km resolution output from each of the abovementioned simulations, to obtain high resolution climate projections over Africa with a spatial resolution of roughly 50 km × 50 km [40]. The topography of the African continent is simulated in the model at roughly 50 km × 50 km. The model topography is smoother than reality, but features such as South Africa's eastern escarpment and the mountainous regions of East Africa are well resolved by the modelled topography. It may be noted that CCAM’s ability to realistically simulate present-day southern African climate has been extensively demonstrated [40,41,42,43,44]. In this study, the simulations were made for the period 1961–2100. The period 1961–1990 is considered as present climate. Three future periods, defined as 2011–2040, 2041–2070 and 2071–2100, are analysed in comparison to the present climate in order to understand the projected changes.

### 2.1. Bias-Correction

In order to project changes in extreme apparent temperature events over Africa, it is useful to first bias-correct the model simulations of temperature and humidity to remove any systematic errors that may otherwise affect the exceedance of critical threshold events. Bias correction compares an observed dataset to the model outputs (*i.e.*, temperature and relative humidity), and then if there is a systematic bias in the model, a correction to the model output is applied across the full data set (*i.e.*, 1965–2100). In this study, the monthly climatologies of the CRU TS3.1 data set (Climatic Research Unit; [45]) were used as the observed climate dataset. The CRU data and CCAM simulations were compared for the period of 1961–1990. The CCAM model simulations were interpolated to the 0.5 degree latitude-longitude grid of the CRU data, to facilitate the generation of gridded bias-corrected simulations [46]. After the model climatologies for each CCAM downscaling were calculated, the simulated daily relative humidity was bias-corrected with a multiplicative factor, whilst the simulated daily maximum temperature underwent an additive correction based on the mean temperature bias. The bias-corrected relative humidity and maximum temperature fields were used in the apparent temperature calculations described in the next section.

### 2.2. Calculating Apparent Temperature

Apparent temperature (AT) aims to describe the ability of the body to cool itself by perspiration and evaporation by accounting for multiple meteorological variables (*i.e.*, temperature, relative humidity and wind speed) in a single index [19,47,48]. AT has been used extensively in previous studies on the association between health and high temperatures (e.g., [6,14,19,27,48,49,50,51,52,53,54,55,56,57,58,59]). Since there are many areas across Africa with high levels of relative humidity that could potentially impact human health, it was deemed important to use an index that accounts for relative humidity.

In this study, AT was calculated following the Australian Bureau of Meteorology equation (Equation (1)): where *Ta* is the dry bulb temperature (°C), *e* is the water vapor pressure (hPa) and *ws* is the wind speed at an elevation of 10 m (m/s) [60,61].

AT = *Ta* + 0.33 × *e* − 0.70 × *ws* − 4.00 (1)

The maximum apparent temperature was calculated daily for each ensemble member. In the results section, the average of the ensemble is analyzed, unless stated otherwise.

### 2.3. Apparent Temperature Thresholds

The projected AT values were grouped in two ways in order to understand the potential for direct heat-related health impacts: (1) number of “hot days” and (2) through the use of symptom bands. Both of these groupings used temperature thresholds from the US National Weather Service symptom chart (Table 1; [62]), since no similar symptom bands are available for Africa.

In order to understand the magnitude of the impact of apparent temperature on human health, it would be vital to use locally derived thresholds. There have been some studies in Africa that have worked to develop a heat-mortality relationship; however, these studies were for only five areas/cities in Africa and did not use a metric such as apparent temperature to include relative humidity considerations [15,16,17,23,63,64]. As there are many areas in Africa that experience high relative humidity, many times coincident with high temperatures, and there is a high variability in the relative humidity levels on the continent, it was deemed important to use an index that accounted for both. Additionally, an advantage of using climate model output over a region such as Africa that has limited spatially comprehensive metrological data [1] is that indices such as AT can be assessed easily through model output, through their calculation from observations. As no comprehensive local relationships exist for Africa, and none that are stratified by the severity of potential symptoms, this international symptom table (Table 1) was used in this study in order to identify the potential for health impacts across the continent. As the AT thresholds in Table 1 are not tested for Africa, this study does not attempt to describe the potential types of negative health impacts that may be experienced or to quantify the impact of AT on human health. Rather, with the limitations of using these thresholds in mind, the aim was to provide a continent-wide overview comparison of the potential risk for health impacts from high AT. In order to provide a comparable assessment across the continent, absolute thresholds were used instead of relative thresholds. An absolute threshold was selected in order to perform a comparable assessment of risk of exposure to constant AT across the continent.

**Table 1:** Apparent temperature thresholds and potential health impacts [62].

Symptom Band	US NWS Classification	Apparent Temperature Range (°C)	US NWS Classified “Effect on Body”
I	Caution	27–32	Fatigue possible with prolonged exposure and/or physical activity
II	Extreme caution	32–39	Heat stroke, heat cramps, or heat exhaustion possible with prolonged exposure and/or physical activity
III	Danger	39–51	Heat cramps or heat exhaustion likely, and heat stroke possible with prolonged exposure and/or physical activity
IV	Extreme Danger	51	Heat stroke highly likely

The AT thresholds in Table 1 were used to understand the projected increase in “hot days” (Hda) by modelling the number of days per year from 1961 to 2100 in the following categories, where *ATmax* is the maximum daily apparent temperature:

- Hda1 = Days where ATmax < 27 °C
- Hda2 = Days where ATmax ≥ 27 °C
- Hda3 = Days where ATmax ≥ 32 °C
- Hda4 = Days where ATmax ≥ 39 °C
- Hda5 = Days where ATmax ≥ 51 °C

The number of days per year in the symptom bands in Table 1 were also modelled and are referred to in the results by the Symptom Band number in Table 1.

In this analysis, it is assumed that ATmax = 27 °C is the threshold where heat starts to have the potential to impact human health. It is also assumed that at higher apparent temperatures the health risk and impact will be greater. It is very likely that different countries and populations will have varying AT thresholds at which health will begin to be affected. However, that information is not well-parameterized for Africa, hence these thresholds were applied. This analysis allows for a common comparison of trends across the continent undertaken for the first time at this scale and geographic spread.

### 2.4. Rate of Increase of Projected Number of Hot Days

A time series of the number of days over each threshold was created for all of Africa. In the time series analysis, the outputs were averaged using an 11-year moving average for the number of days per year in order to decrease the noise in the yearly projections. The average rate of increase for the full time period analyzed was calculated for the median of the ensemble for Hda2, Hda3 and Hda4 thresholds in order to begin to understand the areas on the continent that are projected to experience the most rapid rate of increase in temperature. In this analysis, the average rate was calculated (in days/year) by dividing the change in number of days over the full timescale (*i.e.*, change in days per year in 2095 and 1966) by the change in the number of years over the full timescale (130 years).

The analysis of the number of days in each category (*i.e.*, Hda2, Hda3, *etc.*) from the CCAM output was performed using a Fortran code written for this study. The CCAM model outputs were visualized in Grid Analysis and Display System (GrADS; Institute of Global Environment and Society, USA) and ArcGIS 10 (Esri, Redlands, CA, USA). The Climate Data Operator [65] ensemble percentile function was used to calculate the 90th percentile, 10th percentile and median of the time series of all “hot days” as defined above (*i.e.*, Hda2, Hda3, Hda4).

A Monte Carlo analysis was performed for the time series for Hda2, Hda3 and Hda4 from the median of the ensemble members in order to test for the significance of the projected increasing trend. These trend significance tests were performed using MATLAB code developed for this study that determines the statistical significance of trends at each model grid point through a re-randomization or Monte Carlo test [66]. The time series are re-randomized through resampling with replacement and trends of the new series are subsequently obtained. After 1000 iterations, these trends are ranked and compared with the trends of the original series in order to determine statistical significance at varying levels (e.g., 90%, 95% and 99%).

## 3. Results

### 3.1. Projected Number of Hot Days

The number of days with maximum AT above the AT thresholds in Table 1 (defined as Hda1, Hda2, Hda3, Hda4 and Hda5 in Section 2.3) were modelled and downscaled for Africa per year for 1961–2100 by CCAM. The average number of days per year for each Hda threshold in each specified time period were calculated; and the ensemble average of these average number of days are shown in this section.

**Figure 1:**
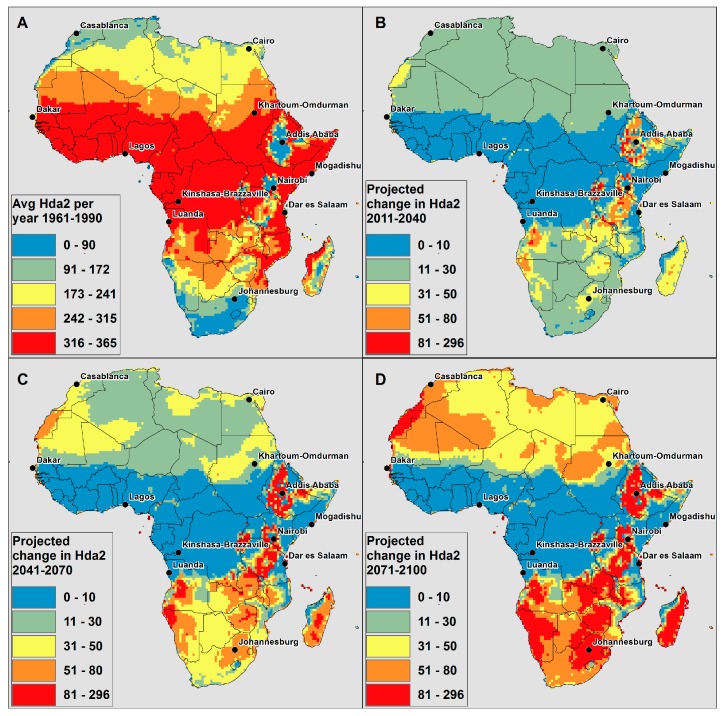
CCAM model derived (**A**) average number of Hda2 per year in present climate; (**B**) projected change in average number of Hda2 per year in 2011–2040 compared to 1961–1990; (**C**) projected change in average number of Hda2 per year in 2041–2070 compared to 1961–1990; (**D**) projected change in average number of Hda2 per year in 2071–2100 compared to 1961–1990.

Figure 1 below shows the average number of Hda2 per year for 1961–1990 (present day climate; Figure 1a), and the average change in number of Hda2 per year for the times slices of 2011–2040 (Figure 1b), 2041–2070 (Figure 1c) and 2071–2100 (Figure 1d). The change is displayed in Figure 1b–d in order to easily highlight the projected impact that climate change will have on the number of hot days. Thus, in order to understand the absolute magnitude of Hda2 projected on average per year in 2011–2040, the change from Figure 1b would need to be added to the number for the average number of days per year in Figure 1a. For example, in present day climate, Johannesburg, South Africa is modelled to have 34.5 Hda2 per year on average for 1961–1990 (blue in Figure 1a). In 2011–2040, Johannesburg is projected to have an increase of 35 Hda2 on average per year (yellow in Figure 1b); this would give a total of 69.5 Hda2 on average per year in 2011–2040.

In general, there is an increase in Hda2 across the continent, which by using ATmax = 27 °C as the threshold for where heat may start impacting health, indicates that there are projected to be more days into the future where health may be impacted by high temperatures. The average number of Hda1 (ATmax < 27 °C) was also modelled (Figure S1). Across the continent, the number of Hda1 decreased, which indicates that the number of days when health is less likely to be impacted by heat are projected to decrease across the continent. This clearly indicates that the potential risk to human health from high AT is projected to increase across the continent.

In Figure 1a, a large part of equatorial Africa and coastal areas (in red) have many Hda2 already in the present day climate; thus it is not possible for there to be a large change in the number of Hda2. However, across the rest of Africa, there is an increase in the average Hda2 per year in each time slice, and the spatial trends in the projected increases are similar across time slices. For example, the areas with the largest projected increases in 2011–2041, such as the high-lying areas of the escarpment from Ethiopia through Tanzania (in orange and red in Figure 1b), are also projected to experience the highest increases in the 2071–2100 time period (in red in Figure 1d). In northern Africa, the increases in Hda2 do not have large spatial variability, while in southern Africa there is more spatial heterogeneity in the projected increases. High spatial resolution regional climate modelling focused on areas with high spatial heterogeneity may be useful in creating tailored projections in order to spatially resolve this heterogeneity.

**Figure 2:**
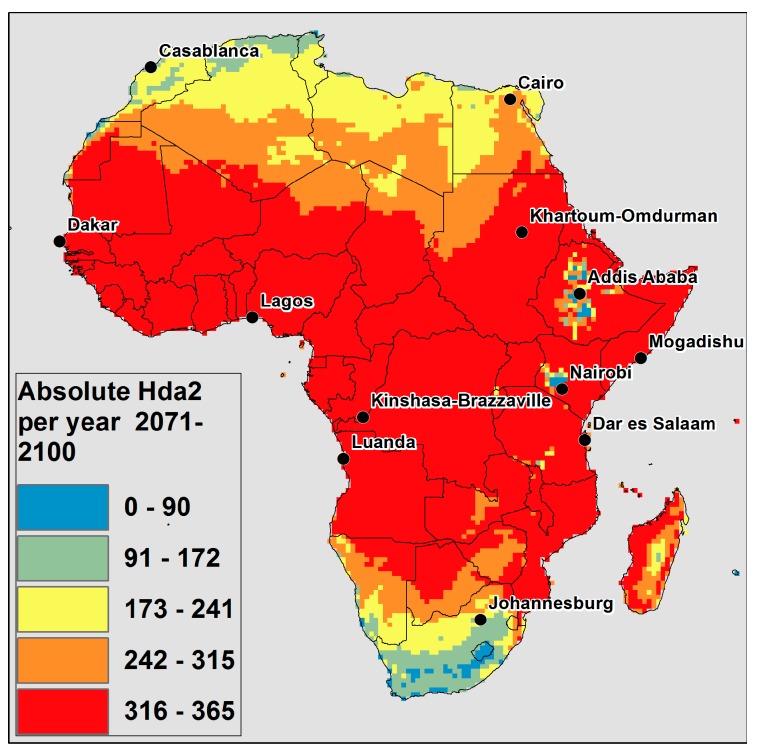
CCAM model outputs for number of Hda2 per year projected in 2071–2100.

Figure 2 displays the average number of Hda2 per year projected for 2071–2100. The color scale is the same as in Figure 1a, which is the average number of Hda2 per year modelled for the current climate. Figure 2 highlights that the spatial extent of Africa that will now have close to every day as Hda2 (in red) has extended from the current climate. In addition, the majority of Africa is projected to have, on average, over 5 months of the year as Hda2 (in yellow, orange and red). While much of South Africa and areas in the East African highlands are modelled to have fewer than 88 Hda2 days per year in the current climate (Figure 1a), very few areas are projected to have fewer than 88 Hda2 per year (in blue) in 2071–2095.

In order to understand the number of days projected across different apparent temperature thresholds, Hda3, Hda4 and Hda5 were analyzed. Applying different thresholds is helpful to understand the potential severity of health impacts, as higher temperatures lead to an increase in mortality. Additionally, the thresholds can be used to understand at what AT threshold different regions are projected to begin to experience increases in “hot days”. For example, as stated above, for the highlands of East Africa, this increase is projected to begin at the Hda2 threshold.

**Figure 3:**
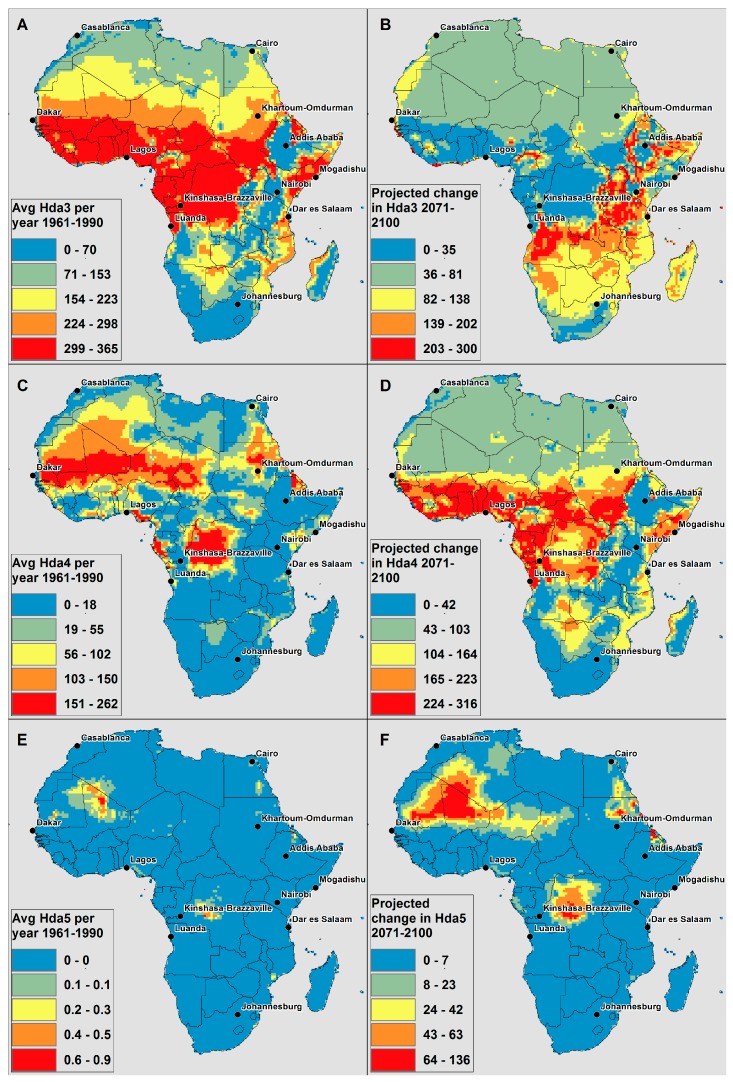
CCAM model derived (**A**) average number of Hda3 per year in present climate (1961–1990); (**B**) change in average number of Hda3 per year in 2071–2100 compared to 1961–1990; (**C**) average number of Hda4 per year in present climate (1961–1990); (**D**) change in average number of Hda4 per year in 2071–2100 compared to 1961–1990; (**E**) average number of Hda5 per year in present climate (1961–1990); (**F**) change in average number of Hda5 per year in 2071–2100 compared to 1961–1990.

Figure 3 displays the number of days on average per year for the present day climate and the projected change in the average number of days per year in the time slice 2071–2100 for Hda3, Hda4 and Hda5 (all time slices for Hda3, Hda4 and Hda5 are displayed in Figures S2–S4 of supplementary data).

There was not a projected decrease in number of days for any of the three thresholds. For Hda3, equatorial Africa, and the western part in particular, there are already numerous days above ATmax = 32 °C, and, as in Hda2 (Figure 1), large increases are not possible. The largest projected increases in Hda3 are seen in a band from Angola, across northern Zambia, southern Democratic Republic of Congo and Tanzania, up across Uganda, Kenya, and Ethiopia (Figure 3b in red). Additionally, there are pockets of projected increases in Hda3 in western Africa (e.g., in Cameroon and Nigeria). In general, these areas are on the border of the areas that already have a large number of Hda3 in the present climate. This suggests that several of these areas displaying large increases in Figure 3b may have many days very close to the threshold in the current climate, and thus the projected increasing temperature will result in shifting a large number of days from just below the Hda3 threshold to above it. This is similar to the increases seen in the high-lying areas of the escarpment in Hda2 in Figure 1.

The spatial extent of areas in Africa with large number of Hda4 days in the present climate is much smaller than Hda2 and Hda3 (Figure 3c). It is at the Hda4 threshold where western equatorial Africa, in particular, is projected to see large increases in number of days. In southern Africa, the northern and eastern Botswana borders with Angola and Zambia, much of Mozambique, and some of the eastern coastal areas are projected to see increases (in yellow and orange in Figure 3d); the rest of the region does not experience many Hda4 in the current climate, and is not projected to see comparatively large increases in number of days by the end of the century.

There are very few Hda5 days in the present climate anywhere in Africa (Figure 3e, note the small scale on the color bar). The spatial extent of areas in Africa that are projected to see increases in Hda5 by 2100 is narrow, with increases in the Democratic Republic of Congo, which is most likely driven by changes in relative humidity, and in the eastern Sahara desert. Previous research has found that the eastern Sahara is one region that is projected to see some of the largest increases in temperature on the continent [67]. Additionally, both of these areas may have many days already close to this threshold, and thus the projected increase moves many days over the AT threshold.

### 3.2. Rate of Increase of Projected Number of Hot Days

**Figure 4:**
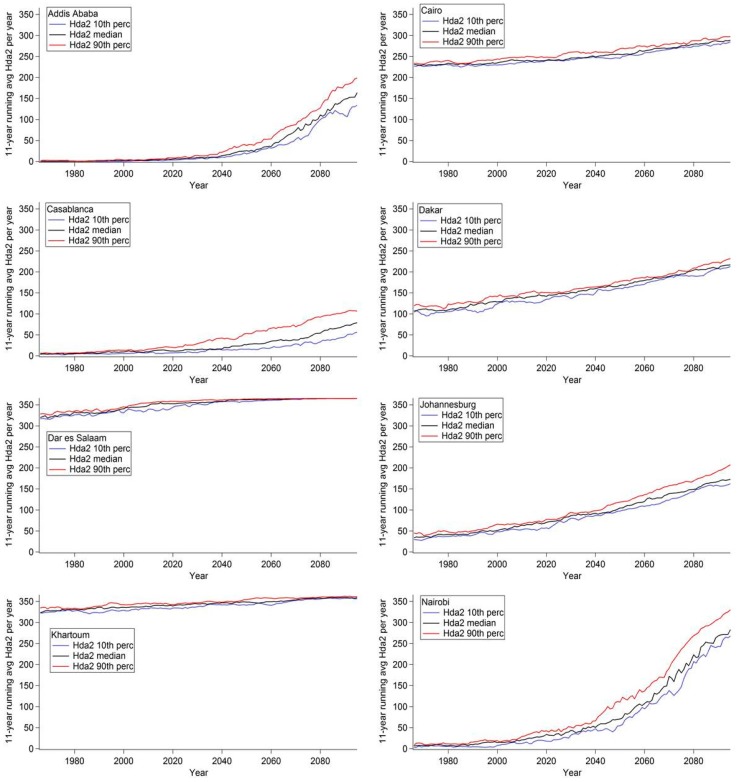
Eleven-year moving average of the number of Hda2 per year in selected cities in Africa. The ensemble 10th percentile (blue), the ensemble median (black) and the ensemble 90th percentile (red) of number of days per year are shown.

**Figure 5:**
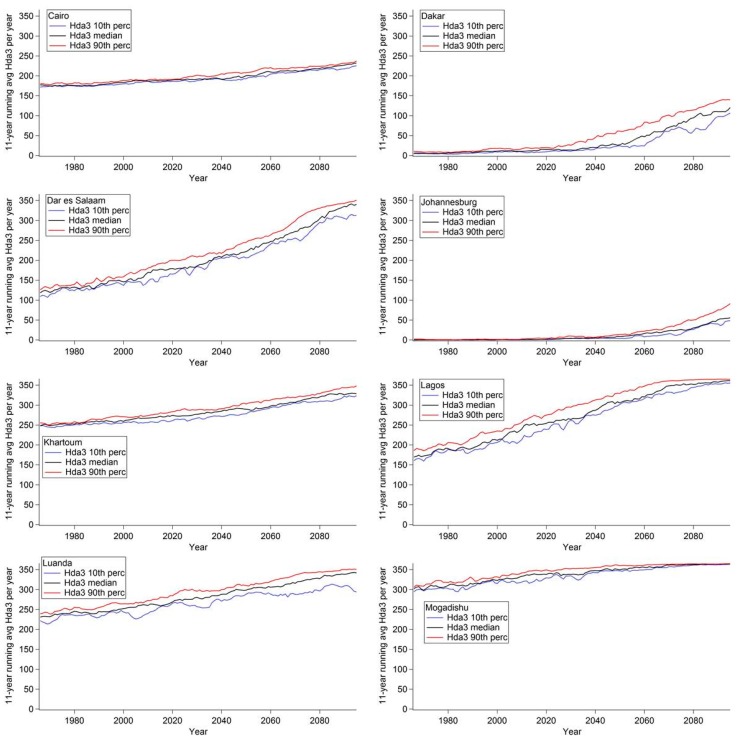
Eleven-year moving average of the number of Hda3 per year in selected cities in Africa. The ensemble 10th percentile (blue), the ensemble median (black) and the ensemble 90th percentile (red) of number of days per year are shown.

**Figure 6:**
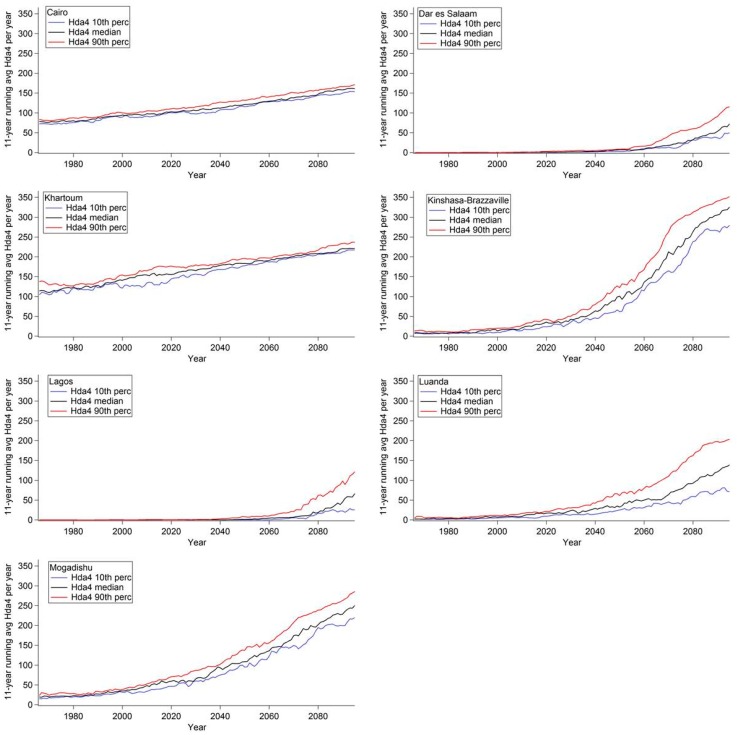
Eleven-year moving average of the number of Hda4 per year in selected cities in Africa. The ensemble 10th percentile (blue), the ensemble median (black) and the ensemble 90th percentile (red) of number of days per year are shown.

In order to begin to understand what areas of Africa are projected to see the largest rates of increase in AT, a time series of the 11-year moving average for the number of hot days for all thresholds (*i.e.*, Hda2, Hda3, *etc.*) was calculated for the full model domain (*i.e.*, Africa). The time series for 12 selected cities are displayed in Figure 4, Figure 5 and Figure 6 below in order to highlight the projected impact on large African cities, as well as to highlight the variability in the projected increases and rate of increases (magnitude and shape) projected for the continent. The 12 cities were selected due to their large and growing populations (Table 2), and for a representative geographical spread of the areas highlighted. Those cities that were analyzed are Lagos, Nigeria; Cairo, Egypt; Kinshasa-Brazzaville conurbation, Democratic Republic of the Congo and Republic of the Congo; Johannesburg, South Africa; Mogadishu, Somalia; Khartoum, Sudan; Dar es Salaam, Tanzania; Casablanca, Morocco; Nairobi, Kenya; Luanda, Angola; Addis Ababa, Ethiopia; and Dakar, Senegal. Not all cities are shown in each figure to ease the viewing of these figures; those cities that were projected to either see no change or a very little change are not shown below, but are included in the supplementary material (Figures S5–S7).

**Table 2:** Population per city as reported by the United Nations Human Settlements Programme [68].

City, Country	Population in 2010	Projected Population in 2020	Projected Population in 2025
Cairo, Egypt	11,031,000	13,254,000	14,740,000
Lagos, Nigeria	10,788,000	15,825,000	18,857,000
Kinshasa-Brazzaville conurbation, Democratic Republic of the Congo and Republic of the Congo	9,972,000	14,396,000	16,899,000
Luanda, Angola	4,790,000	7,555,000	8,924,000
Khartoum, Sudan	4,516,000	6,018,000	7,090,000
Johannesburg, South Africa	3,763,000	4,421,000	4,732,000
Nairobi, Kenya	3,237,000	4,939,000	6,143,000
Dar es Salaam, Tanzania	3,415,000	5,677,000	7,276,000
Casablanca, Morocco	3,009,000	3,580,000	3,911,000
Addis Ababa, Ethiopia	2,919,000	3,881,000	4,705,000
Dakar, Senegal	2,926,000	4,227,000	5,064,000
Mogadishu, Somalia	1,426,000	2,693,000	3,309,000

Figure 4 displays the 11-year moving average of Hda2 per year, Figure 5 displays the 11-year moving average of Hda3 per year, and Figure 6 displays the 11-year moving average of Hda4 per year for selected African cities. In each graph, three model analyses were plotted for each threshold as a function of time; the ensemble 10th percentile (blue), the ensemble median (black), and the ensemble 90th percentile of the number of days per year (red). Figure 4, Figure 5 and Figure 6 show that for each city the three outputs show the same trends, however, there is a difference in magnitude and this difference varies depending on the city.

In Figure 4, the plots for Dar es Salaam and Khartoum highlight examples of cities that are projected to have a small change in the number of Hda2, as those cities already experience a large number of Hda2 in today’s climate. Addis Ababa and Nairobi see a non-linear rate of increase in the projected number of days, with the rate increasing in the latter part of the century. Both of these cities are in the area in red in Figure 1, which indicates that they are projected to see the largest increases in Hda2; Figure 4 also highlights the projected magnitude of the rate of change and how it is projected to change in the time period modelled. The other cities highlighted, namely Johannesburg, Dakar, Cairo and Casablanca, also see increases in Hda2, however, the shape of the time series is closer to linear than the time series for Nairobi and Addis Ababa.

In Figure 5, Casablanca, Nairobi and Addis Ababa are not shown as they are projected to see very small increases in Hda3 compared to the other cities studied, which lead to few Hda3 projected in 2095 (the ensemble 90th percentile value of Hda3 < 33 days in 2095). Thus, these cities are projected to see increases in days with ATmax ≥ 27 °C (*i.e.*, Hda2) but not in days with the threshold of ATmax ≥ 32 °C (*i.e.*, Hda3); this indicates that the largest projected increases are occurring in the AT range of 27 °C ≤ ATmax ≤ 32 °C.

Johannesburg and Dakar are both projected to have increases in Hda3 from very few days in the current climate, with Dakar projected to see the increases earlier in the century than Johannesburg. The other cities are projected to see steady increases in Hda3, and all, except for Cairo, are projected to see increases in Hda3 until almost every day is projected to be an Hda3 by the end of the century.

In Figure 6, Kinshasa-Brazzaville is shown for the first time, as at the Hda2 and Hda3 thresholds the conurbation already had a large number of days in the current climate and thus could not see an increase into the future (Figures S5 and S6). For Hda4, this conurbation is projected to experience a large non-linear increase. Cairo and Khartoum have a steady increase in the number of days. It should be noted that across Figure 4, Figure 5 and Figure 6, for Cairo, and across Figure 5 and Figure 6 for Khartoum, the shape of the time series was very similar showing a very steady increase. Many of the other cities showed a variable rate of increasing days at one of the thresholds (*i.e.*, Nairobi in Figure 5, Kinshasa-Brazzaville in Figure 6); however, neither Cairo nor Khartoum are projected to see this type of increase. This difference in the shape of the time series in different cities, which has implications for the rate of increase of the number of hot days, highlights the variability of the projected impact of climate change on apparent temperature across the continent. This reiterates the need for high resolution regional climate projections that provide projections on smaller areas with a finer spatial scale which can provide finer details of spatial differences in temperature and the drivers of the heterogeneity. Further investigation of the meteorological reasons driving the differences in the rate of change for these cities fall beyond the scope of this paper.

A Monte Carlo analysis was performed for the time series for Hda2, Hda3 and Hda4 from the median of the ensemble members (e.g., black lines in Figure 4, Figure 5 and Figure 6) in order to test for the significance of the increasing trends seen. Across the full model domain, the increasing trend was significant at the 99% significance level, except for areas that saw no change in the number of days either because the area already has 365 days per year at or above that threshold in the current climate (e.g., Lagos in Hda2) or that the area does not currently have any days at or above a threshold and is not projected to have any increase at that level in the future (e.g., East African Highlands in Hda4). Thus, at all areas when an increase is projected, the increase was determined to be statistically significant.

**Figure 7:**
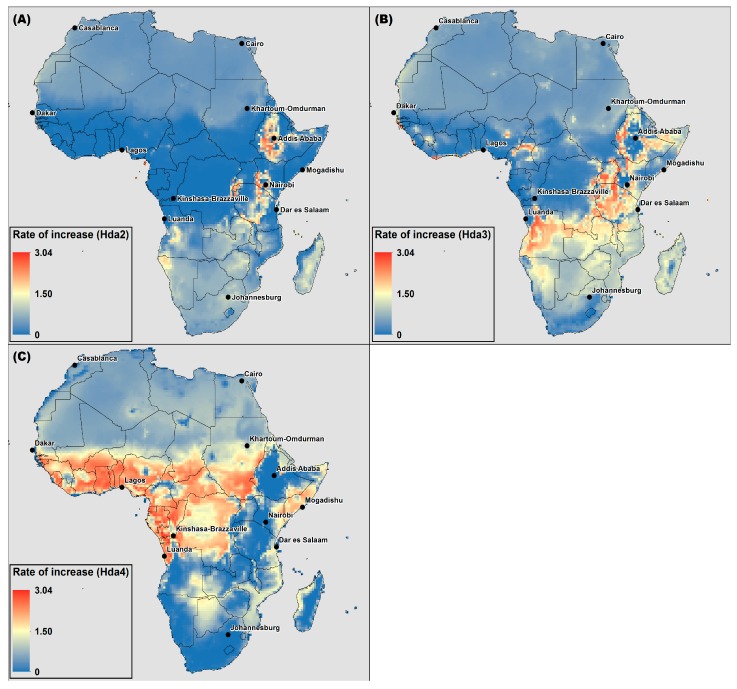
The average rate of increase of the 11-year moving average of (**A**) Hda2; (**B**) Hda3; (**C**) Hda4 for the median ensemble member for 1966–2095.

In order to analyze the rate of increase across Africa, the average increase in days per year over the full timescale of the calculated 11-year moving averages (*i.e.*, 1966–2095) was calculated for Hda2, Hda3 and Hda4 and is shown in Figure 7. An average increase was selected for this analysis in order to provide a comparable analysis of the rate of increase across the entire domain. In Figure 7a, the average rate of increase for Hda2 is displayed. In this figure, the highlands in East Africa are projected to see the largest average rate of increase (red). Nairobi is projected to see an average rate of increase of 1.98 Hda2 per year, and Bujumbura, Burundi is projected to see one of the higher projected average rates of increase at 2.36 Hda2 per year. In comparison, Johannesburg, South Africa, which also is projected to see large increases in the number of Hda2 (Figure 1) is projected to see an average rate of increase of 1.10 Hda2 per year. While the ability of populations to acclimatize to new climatic regimes is not well-understood, it may be assumed that a higher rate of temperature increase can lead to a higher risk of negative health impacts. This analysis highlights that while both the East African highlands and areas in southern Africa are projected to see large increases in Hda2, the average rate of increase is projected to be higher in the highlands, and thus this area may be a “hot spot” with an increased risk of negative health impacts from heat.

Figure 7b highlights the average rate of increase in Hda3 over the time period studied. For this threshold, many areas surrounding the East African Highlands, as well as parts of Angola and Nigeria and Cameroon are projected to experience the highest rates of increase, with smaller average increases projected for southern Africa (in yellow) and northern Africa (in light blue). In Figure 7c, much of western equatorial Africa is projected to experience the largest average rate of increase in Hda4. The rate of increase in this area according to Figure 7a,b was low because in the current climate there already are many Hda2 and Hda3, and thus there is little potential for increase and thus there is a small increase in the projected number of days. Conversely, the rate of increase in, for example, the highlands of East Africa in Hda4 (Figure 7c) is low because there are very few or no days in this threshold in the current climate, and none projected during the timescale analyzed in this study. Thus, it is critical to analyze Figure 7 together with the Figure 1 and Figure 3 in order to interpret the results of the average rate of increase.

### 3.3. Symptom Bands

**Figure 8:**
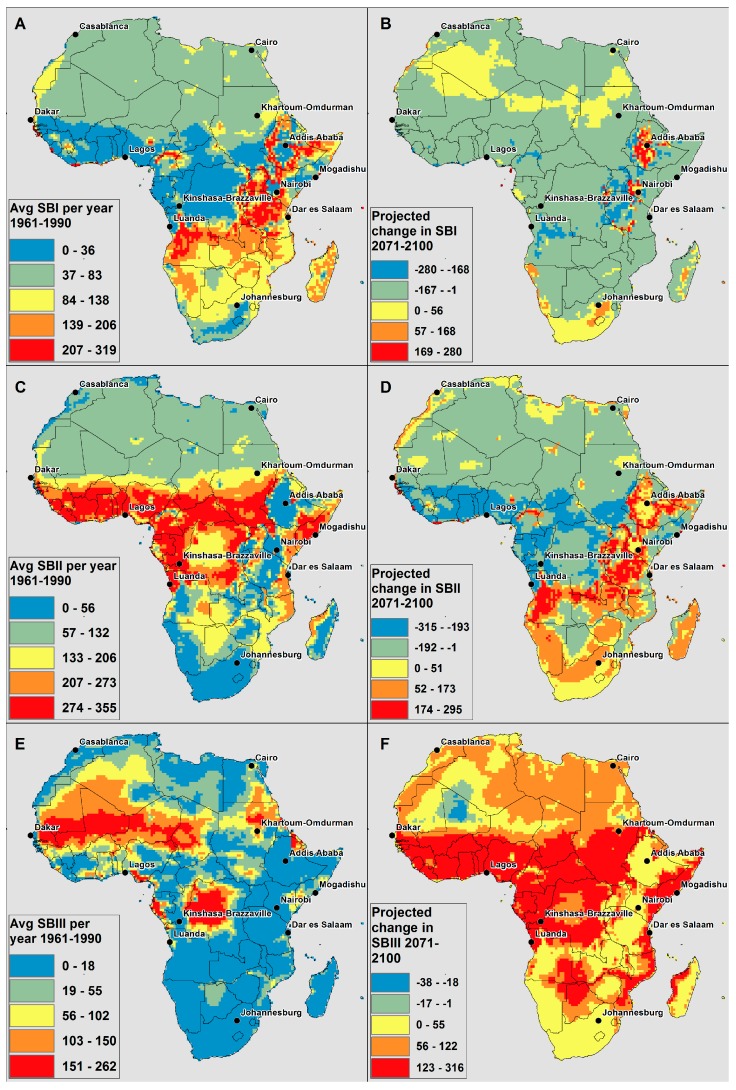
CCAM model derived (**A**) average number of Symptom Band I days per year in present climate (1961–1990); (**B**) change in average number of Symptom Band I days per year in 2071–2100 compared to 1961–1990; (**C**) average number of Symptom Band II days per year in present climate (1961–1990); (**D**) change in average number of Symptom Band II days per year in 2071–2100 compared to 1961–1990; (**E**) average number of Symptom Band III days per year in present climate (1961–1990); (**F**) change in average number of Symptom Band III days per year in 2071–2100 compared to 1961–1990.

The assessment of AT thresholds is helpful for understanding the trends in increasing temperature across Africa with respect to potential temperature-related health impacts. In order to better understand in which AT range the largest increases are seen, the number of days within each symptom band was analyzed. Figure 8 shows the ensemble average of the average number of days per year within each Symptom Band (see Table 1) for 1961–1990 (present day climate (left)) and the change (increasing or decreasing) in average days per year for 2070–2100 (right) (all time slices are shown in Figures S8–S10). The ensemble average of the average number of days per year in the 30 year period is shown in Figure 8.

In the projections of number of days within each Symptoms Band in Figure 8, there are areas of Africa that will see decreases in days across all Symptom Bands. The decreases in days in the Symptom Bands are not because there are fewer “hot days”, but rather that days are moving up to more severe Symptom Bands.

For Symptom Band I (Figure 8a,b), the majority of Africa is projected to see decreases in the average number of days per year in this band (blue and green in Figure 8b); the highlands in East Africa, and parts of northern and southern Africa are the only areas projected to see increases (yellow, orange and red in Figure 8b). For Symptom Band I, it appears that there is a difference in the areas that have few of these days in the current climate (blue in Figure 8a) due to having either generally warmer temperatures (*i.e.*, tropical Africa) or generally cooler temperatures (*i.e.*, parts in southern Africa and the highlands in East Africa). The tropical African areas are projected to see decreases in the number of days in this Symptom Band, while the latter areas are projected to see increases.

In Figure 8c, it can be seen that much of the area in equatorial Africa that was in blue in Figure 8a is now in red; the red in Figure 8c indicates a large number of days in Symptom Band II in the current climate. Many parts of southern Africa and the highlands of East Africa have few days in Symptom Band II in the current climate. Figure 8d shows that while large parts of southern Africa are projected to see increases in days in Symptom Band II (yellow, orange and red), the majority of northern and equatorial Africa is projected to see decreases (blue and green).

In the current climate, only a small spatial extent of the continent has many days in Symptom Band III (Figure 8e), and the greatest increases are projected in equatorial Africa (Figure 8f). Only a small part of the Western Sahara is expected to see decreases in Symptom Band III (39 °C ≤ ATmax < 51 °C). This is also a region projected to see increases in Hda5 (ATmax ≥ 51 °C, Figure 3f) and thus this decrease in Symptom Band III appears to be due to a shifting of the days to a higher temperature and a more severe Symptom Band.

## 4. Discussion and Conclusions

High ambient temperatures can have large negative health impacts, but few studies have been carried out in Africa to understand and quantify the relationship between temperature and health [15,16,17,23,63,64]. As there are few temperature-health epidemiological studies in Africa, it was not possible to use locally-derived temperature thresholds for when health may begin to be affected by heat. This is a limitation of the study, and this analysis can be refined as more dose-response relationships are developed for Africa. Future research should focus on developing a comprehensive set of relationships to use for African populations in order to quantify the impact of increasing temperatures and apparent temperatures on human health.

This current study aimed to describe the potential risk to human health from direct exposure to high temperatures as a result of climate change across Africa. In general, the continent is projected to see increases in the number of days when health may be adversely affected by heat due to climate change, as well as the “shifting” of days into a more severe Symptom Bands than in the current climate.

In the current climate, the differences in the number of days at the different thresholds in different cities is informed by the altitude, the line of latitude, proximity to the ocean and the associated ocean temperatures, as well as hemispheric positioning. In the climate projections of the future climate, at different apparent temperature thresholds, different areas across Africa can be identified as “hot spots” (*i.e.*, areas projected to see large increases in “hot days”). There are many possible drivers for this variation. In some areas, the apparent temperature threshold where the increases begin may be dependent on the number of days already close to that threshold in the current climate or close to the climate. For example, the highlands of East Africa were identified as an area that is projected to experience large increases in Hda2, though not in Hda3, Hda4 or Hda5. This large increase may suggest that the highlands have many days in the current climate close to the Hda2 threshold, which the increased warming moves over the threshold apparent temperature. This area in particular is very close to areas with many Hda2 already, but most likely due to the higher altitude do not experience many Hda2 in the current climate. Additionally, the magnitude of warming is not projected to be uniform across the continent, and thus this variation in “hot spots” can also be due to the spatial heterogeneity in the projected warming trends. For example, previous research has indicated that both sub-tropical northern and sub-tropical southern Africa are projected to experience the largest increases in temperature on the continent, which could then lead to “hot spots” [67]. Finally, this study utilizes apparent temperature, which considers temperature and the relative humidity. Thus, the variation in the “hot spots” may also be driven by changes in the projected impact of climate change on relative humidity. Additional regional climate modelling with a smaller spatial domain and even higher resolution could provide the necessary information in order to understand the importance of these drivers in specific areas.

The ability of people to acclimatize to rapid and sustained changes in temperature such as those projected by climate change is not known [11,25]. Previous studies have found evidence of decreasing mortality rates from high temperatures in the past century that may point to adaptation of populations to high temperatures [30,31,32,33,34,35], However, it must be noted that it is possible that temperature increases due to climate change may be greater for Africa in the future than they have been in the past [69], and thus the adaptation levels that may have occurred in the past in industrialized areas may not necessarily be wholly applicable to communities in Africa in a changing climate. Thus, while some adaptation may occur, it can be assumed that the populations that live in the areas that are projected to see faster increases in temperature may be more at risk of negative health impacts from high temperatures compared to those areas with slower rates of temperature increase. The analysis of the average rate of change for the ensemble median of the 11-year moving average time series highlighted the variability of the average rate of increasing temperatures across the continent and across thresholds. In addition, the analysis of the rate of increasing temperatures assisted in identifying areas, such as the East African highlands, where health may be at increasing risk due to both large increases in the numbers of hot days, and due to the high rate of increasing hot days (*i.e.*, high day/year increase). The average rate over the full time period studied was used in order to provide a comparison across the full model spatial domain and across different thresholds. As many of the areas are projected to see increases in hot days that are not linear, and that some areas that are projected to start to experience increases in hot days mid-century (e.g., Johannesburg, South Africa in Figure 5) a more detailed local analysis of the rate of change (e.g., rate of change per decade) is needed to understand the rate of change and how it is projected to change into the future.

Currently, epidemiological studies of heat-health focus on specific high temperature events, but very little is known about the health impacts of living at constant high temperatures that have increased relatively rapidly (*i.e.*, in the span of one century) as is projected for climate change. As is highlighted in this study, many areas in Africa are projected to see large increases in “hot days” which may lead to longer stretches of consecutive hot days; thus additional research on the chronic effects of heat exposure are necessary. Such findings could assist in understanding the magnitude of the health impacts from increasing temperatures in Africa due to both long and short term exposure to high temperatures.

There is very little information on the magnitude of the impact of heat on health in Africa in the current climate. This research highlights that increasing apparent temperatures due to climate change will likely be a risk to human health in Africa in the future. In addition, this research highlights the variability of the changes in apparent temperature which can lead to variability in the risk to human health that are projected for Africa. While the whole continent is projected to see increases in the number of days where health may be impacted by heat, the magnitude and rate of this change, together with the threshold apparent temperature where the change begins, is highly variable across the continent. Thus, studies focused on one country/area are needed to develop area-specific and tailored information on the potential health impact of increasing temperatures.

Climate services that provide climate information and projections, such as those shown here, can provide the health sector with information to better plan for adaptation to the projected increases in apparent temperature to reduce the associated health impacts. Health stakeholders can use such outputs to understand the potential risk to human health, the projected magnitude and rate of change of the increasing risk, as well as identify geographic areas of concern. The identification of “hot-spot” areas is an important step to assist in prioritizing risks in public health. Additionally, primary data collection is key to understand the historical health impacts from high temperatures, including the development of local temperature–mortality relationship [70]. The historical information from primary data collection and projected future risks from regional climate modelling can help decision makers to understand if high temperatures are, and will continue to be, a risk to health as well as the potential magnitude of the health impact. If high temperatures are considered to be a high risk, then an assessment of adaptation options can be performed, such as through following the framework in Ebi and Burton [71]. This framework assists in screening potential adaptation options including factors such as their technical feasibility, degree of effectiveness, environmental acceptability, economic efficiency, social and legal acceptability and compatibility, and the available human and financial capacity of the area.

An adaptation effort that has been used successfully to mitigate the impacts of high temperatures on public health, and is identified as a key adaptation measure to protect public health from such risks into the future, is a heat-health action plan, or Heat Alert and Response Systems (HARS) [59,70,72,73,74,75,76,77,78,79,80]. Through an assessment of existing HARS across countries, Health Canada has created a framework for the development and implementation of HARS. The core elements in HARS are (i) community mobilization and engagement through a lead organization to prepare the community for the upcoming heat season, as well as to identify any needs in the community; (ii) an alert protocol that will be followed, which begins with identification and forecasting of weather conditions that may have an impact on health, and the communication of when this will happen (*i.e.*, through weather forecasts) to key leading organizations and stakeholders; (iii) a community response plan that implements the agreed upon interventions and actions that will be implemented to mitigate health impacts during a heat alert period; (iv) communication plan that provides advice on individual actions that can protect health from high temperatures; (v) an evaluation plan, to assess the HARS and its performance. The WHO [80] highlights that a HARS does not need the creation of a new system, but should build upon existing systems as much as possible in order to incorporate lessons learned from previous disaster response and management systems and activities, and to not over-burden resource-poor areas in need of such plans. However, before an HARS can be developed, there is local research that is needed to create an effective and tailored HARS plan. For example, in developing an alert protocol, research is critical to identify the climatic conditions (e.g., temperature, AT) where health may be impacted by health together with the ability to forecast such conditions by the local meteorological agency who provides operational forecasts [77,81]. In addition, the assessment framework by Ebi and Burton [71] can be re-applied to develop and assess the needed interventions and actions that should be implemented in the community response plan and the communication plan.

This study provides one key piece of the puzzle in mitigating the health impacts from climate change in Africa. Through using these climate projections, “hot-spot” area can be identified. The projections generated in this study were for all of Africa; however, the same type of regional climate modelling, covering a smaller spatial area, with higher spatial resolution, and tailored to the specific area, can provide the climate information needed for area-specific analyses. The climate change projections from this analysis are available freely from the CSIR (please contact the corresponding author for details). Additional high-resolution climate change projections for Africa from CCAM, using emission scenarios from the IPPC Fifth Assessment Report and consistent with the experimental design of the Coordinated Regional climate Downscaling Experiment (CORDEX), are also available.

## Supplementary Files

Supplementary File 1
