# Supplementary material for: Regional Projections of Extreme Apparent Temperature Days in Africa and the Related Potential Risk to Human Health
Source: Int J Environ Res Public Health. 2015 Oct 12;12(10):12577–604. doi: 10.3390/ijerph121012577 (PMC4626987; doi:10.3390/ijerph121012577)
Supplement: Supplementary File 1 [file ijerph-12-12577-s001.pdf]

Supplementary Results for Regional Projections of Extreme Apparent-Temperature Days in Africa and the Related Potential Risk to Human Health

The model results of the number of days where ATmax < 27 °C (Hda1) for Africa projects decreases in these days across the continent (Figure S1). This indicates, using ATmax = 27 °C as the threshold for apparent temperature where health can be impacted by heat, that the potential risk to human health from high temperatures will increase into the future due to climate change.

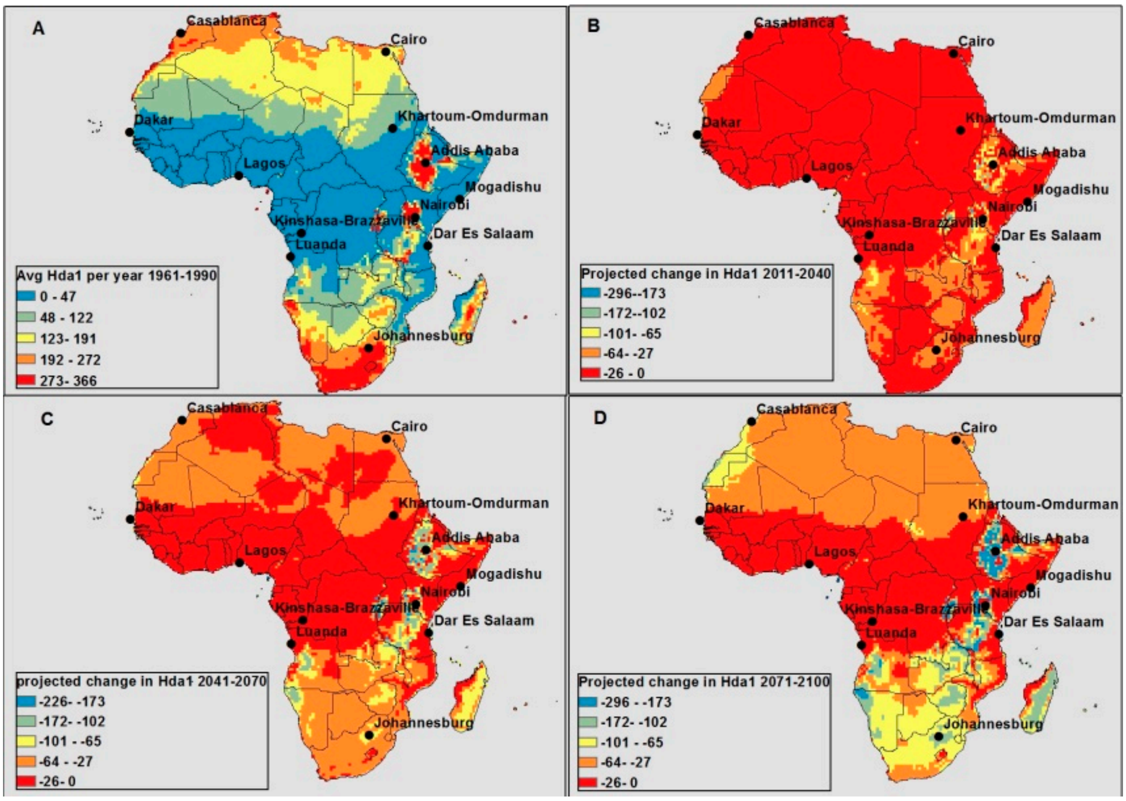

Figure S1. CCAM model outputs for (**A**) average number of Hda1 per year in present climate, (**B**) projected change in average number of Hda1 per year in 2011–2040 compared to 1961–1990, (**C**) projected change in average number of Hda1 per year in 2041–2070 compared to 1961–1990, (**D**) projected change in average number of Hda1 per year in 2071–2100 compared to 1961–1990.

Figure S2 below displays all four time periods for Hda3, Figure S3 all four time periods of Hda4, and Figure S4 all four time periods for Hda5.

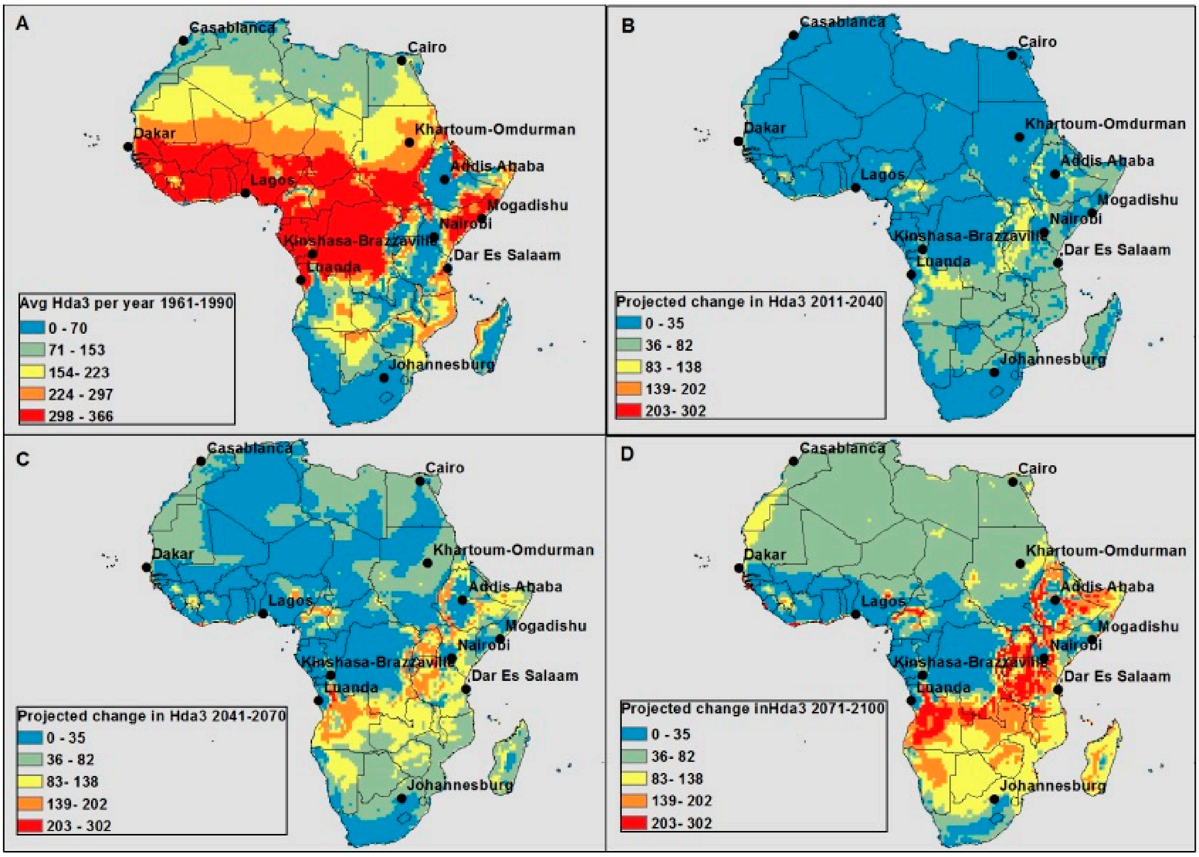

Figure S2. CCAM model outputs for (**A**) average number of Hda3 per year in present climate, (**B**) projected change in average number of Hda3 per year in 2011–2040 compared to 1961–1990, (**C**) projected change in average number of Hda3 per year in 2041–2070 compared to 1961–1990, (**D**) projected change in average number of Hda3 per year in 2071–2100 compared to 1961–1990.

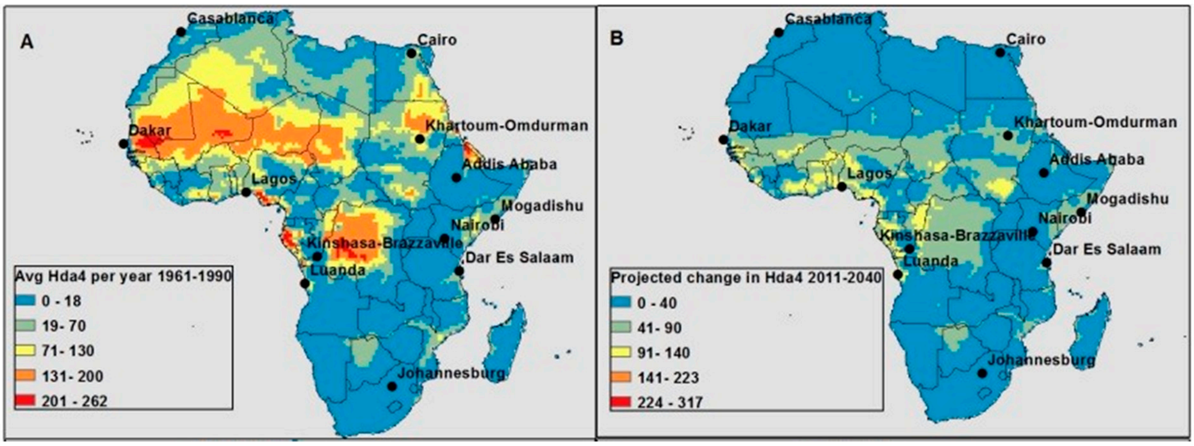

Figure S3. *Cont.*

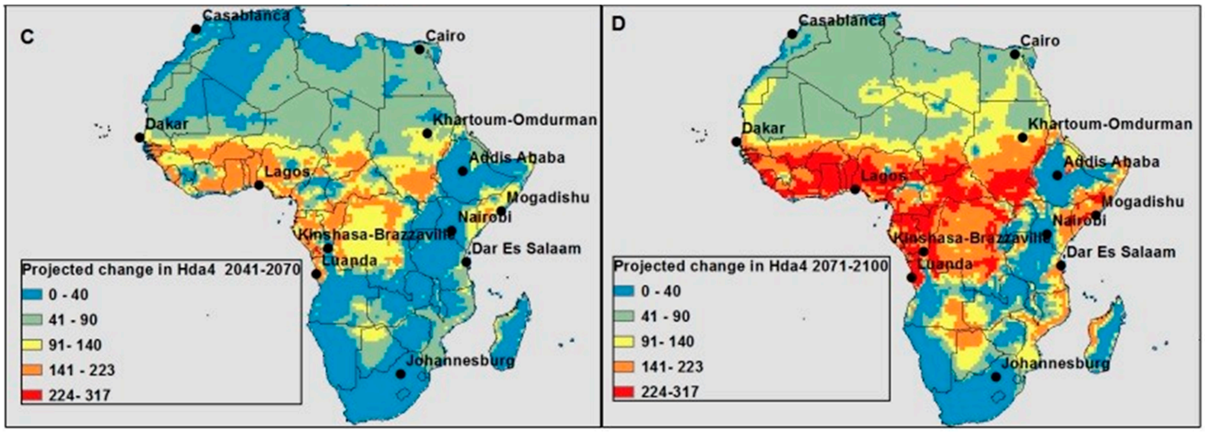

Figure S3. CCAM model outputs for (**A**) average number of Hda4 per year in present climate, (**B**) projected change in average number of Hda4 per year in 2011–2040 compared to 1961–1990, (**C**) projected change in average number of Hda4 per year in 2041–2070 compared to 1961–1990, (**D**) projected change in average number of Hda4 per year in 2071–2100 compared to 1961–1990.

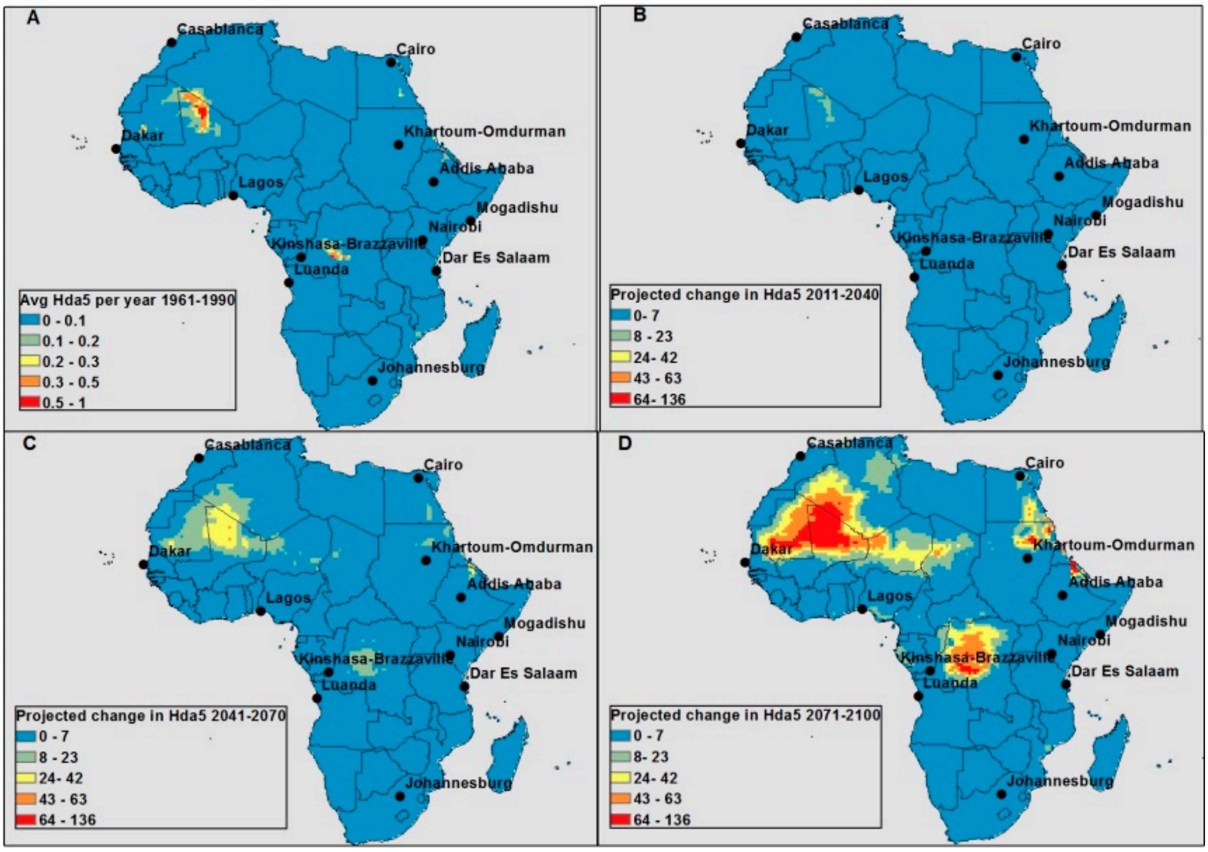

Figure S4. CCAM model outputs for (**A**) average number of Hda5 per year in present climate, (**B**) projected change in average number of Hda5 per year in 2011–2040 compared to 1961–1990, (**C**) projected change in average number of Hda5 per year in 2041–2070 compared to 1961–1990, (**D**) projected change in average number of Hda5 per year in 2071–2100 compared to 1961–1990.

Figure S5 displays the 11-year moving average of the number of Hda2 per year for the selected cities in Africa that were not shown in the manuscript.

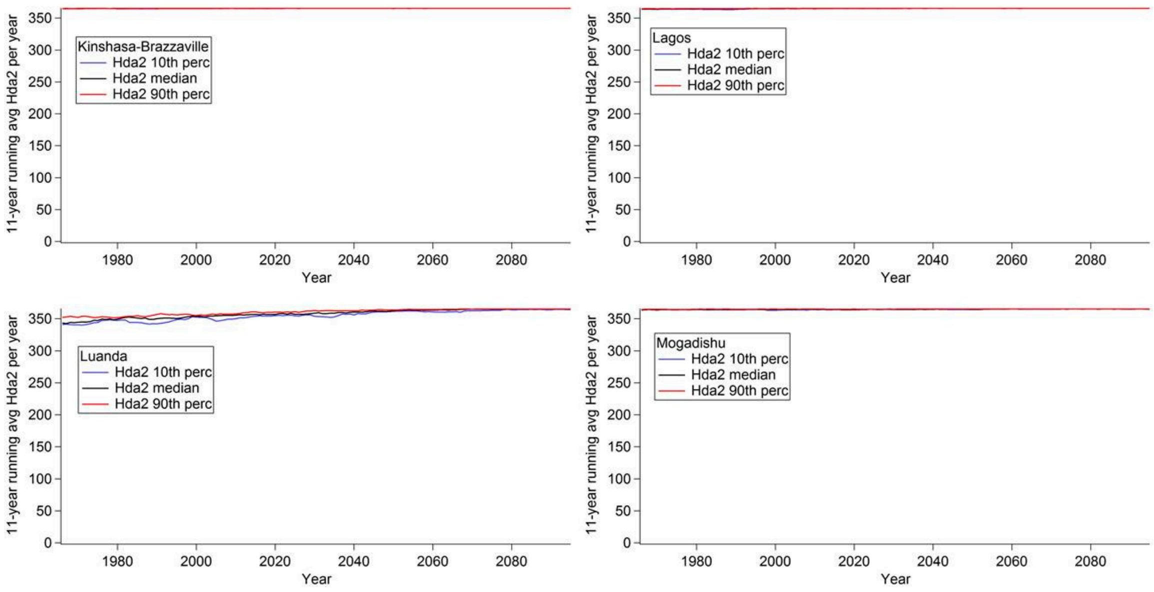

Figure S5. Eleven-year moving average of the number of Hda2 per year in selected cities in Africa. The ensemble 10th percentile (blue), the ensemble median (black) and the ensemble 90th percentile (red) of number of days per year are shown.

Figure S6 displays the 11-year moving average of the number of Hda3 per year for the selected cities in Africa that were not shown in the manuscript.

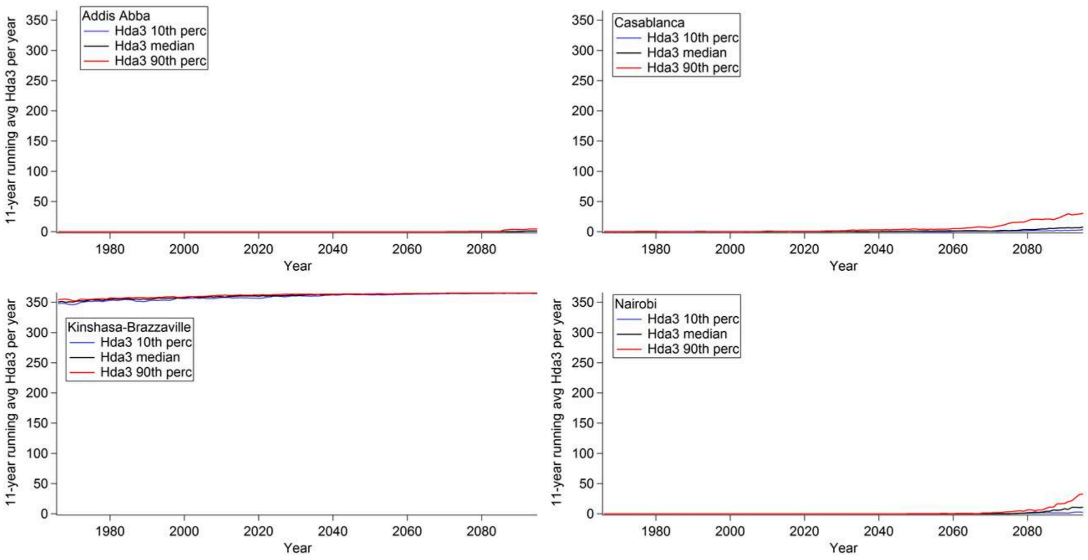

Figure S6. Eleven-year moving average of the number of Hda3 per year in selected cities in Africa. The ensemble 10th percentile (blue), the ensemble median (black) and the ensemble 90th percentile (red) of number of days per year are shown.

Figure S7 displays the 11-year moving average of the number of Hda4 per year for the selected cities in Africa that were not shown in the manuscript.

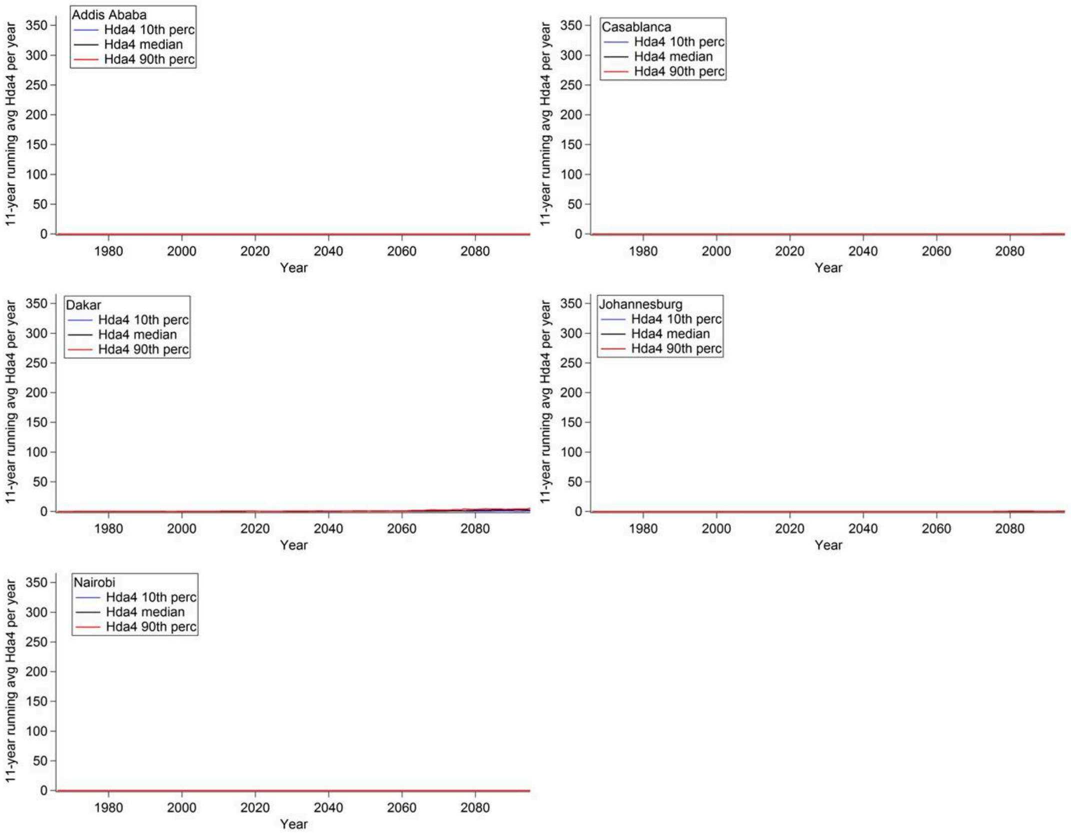

Figure S7. Eleven-year moving average of the number of Hda4 per year in selected cities in Africa. The ensemble 10th percentile (blue), the ensemble median (black) and the ensemble 90th percentile (red) of number of days per year are shown.

Figure S8 below displays all four time slices for Symptom Band I, Figure S9 for all four time slices of Symptom Band II, and Figure S10 for all four time slices for Symptom Band III.

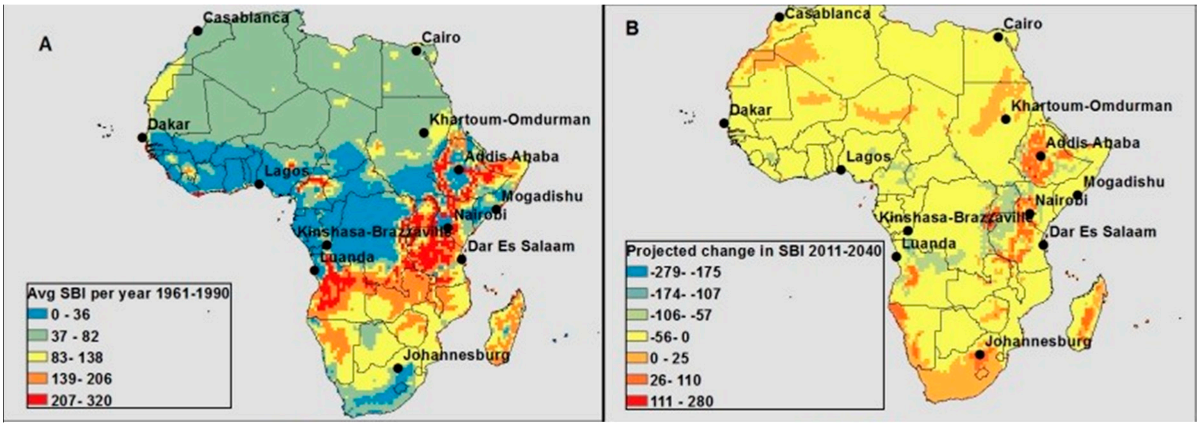

Figure S8. *Cont.*

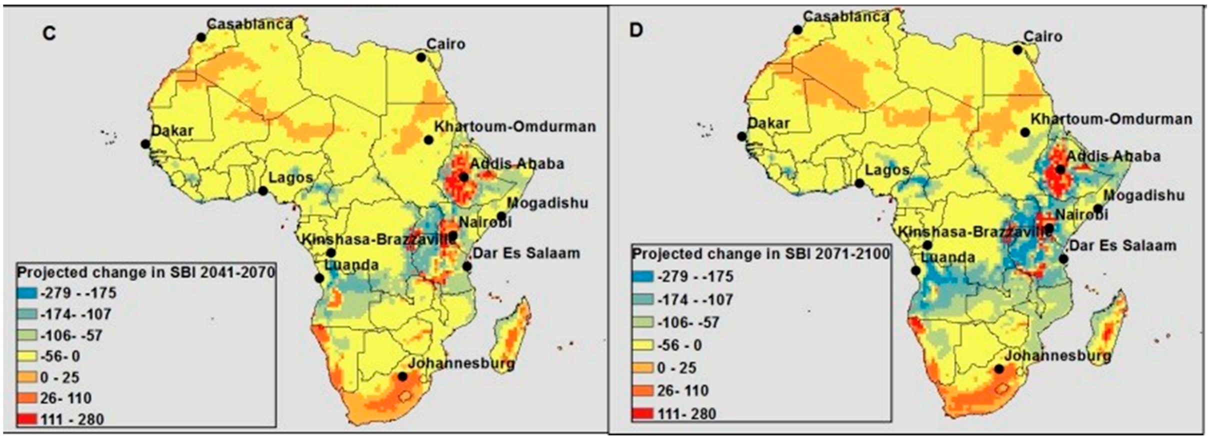

Figure S8. CCAM model outputs for (**A**) average number of days in Symptom Band I per year in present climate, (**B**) projected change in average number of days in Symptom Band I per year in 2011–2040 compared to 1961–1990, (**C**) projected change in average number of days in Symptom Band I per year in 2041–2070 compared to 1961–1990, (**D**) projected change in average number of days in Symptom Band I per year in 2071–2100 compared to 1961–1990.

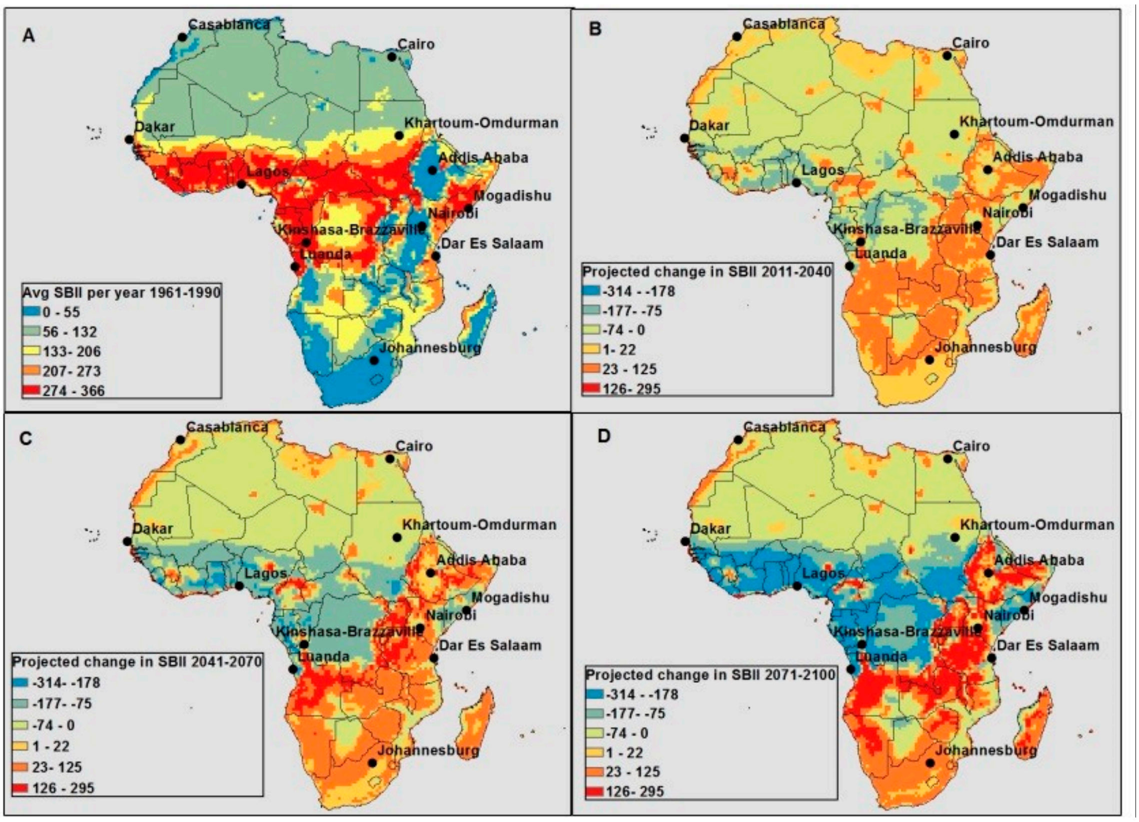

Figure S9. CCAM model outputs for (**a**) average number of days in Symptom Band II per year in present climate, (**b**) projected change in average number of days in Symptom Band II per year in 2011–2040 compared to 1961–1990, (**c**) projected change in average number of days in Symptom Band II per year in 2041–2070 compared to 1961–1990, (**d**) projected change in average number of days in Symptom Band II per year in 2071–2100 compared to 1961–1990.

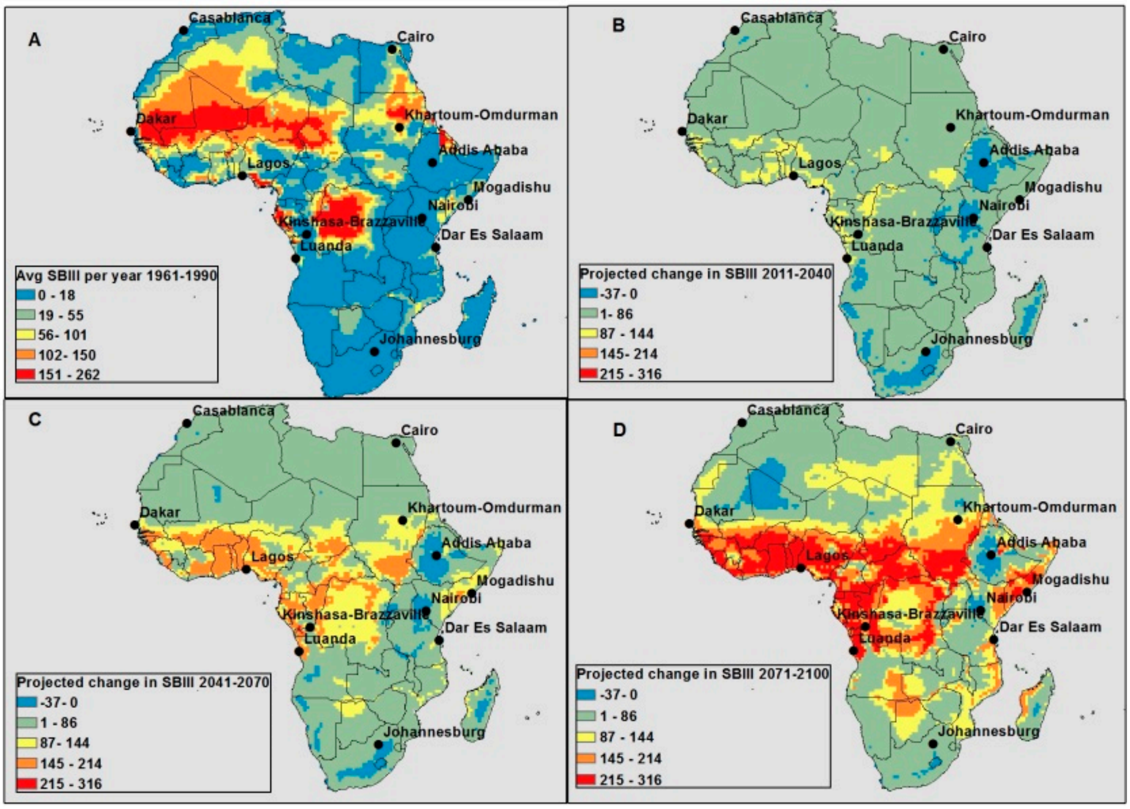

Figure S10. CCAM model outputs for (**a**) average number of days in Symptom Band III per year in present climate, (**b**) projected change in average number of days in Symptom Band III per year in 2011–2040 compared to 1961–1990, (**c**) projected change in average number of days in Symptom Band III per year in 2041–2070 compared to 1961–1990, (**d**) projected change in average number of days in Symptom Band III per year in 2071–2100 compared to 1961–1990.

© 2015 by the authors; licensee MDPI, Basel, Switzerland. This article is an open access article distributed under the terms and conditions of the Creative Commons Attribution license (http://creativecommons.org/licenses/by/4.0/).
